# Minimally invasive dual incision with mini plate internal fixation improves outcomes over 30 months in 20 patients with Sanders type III calcaneal fractures

**DOI:** 10.1186/s13018-020-01644-3

**Published:** 2020-05-05

**Authors:** Jianming Chen, Zhongmeng Yang, Changwang Kong, Shijun Wei

**Affiliations:** 1 Department of Trauma Orthopaedics, Ningbo No.6 Hospital, Ningbo, Zhejiang Province People’s Republic of China; 2 Department of Orthopaedics, General Hospital of Central Theater Command (Wuhan General Hospital of Guangzhou Command, previously), No. 627, Wuluo Road, Wuhan, 430030 Hubei Province People’s Republic of China; 3 Department of Orthopaedics, The Fifth Affiliated Hospital of Sun Yat-Sen University, Zhuhai, Guangdong Province People’s Republic of China

**Keywords:** Mini plate internal fixation, Minimally invasive dual incision, Sanders type III calcaneal fractures, Calcaneal geometry, Wound infection

## Abstract

**Background:**

Calcaneal Sanders type III or higher fractures traditionally have been treated with open reduction and internal fixation (ORIF); however, ORIF has associated complications. We investigated a combination of minimally invasive dual incision and internal fixation using mini plates for treating Sanders type III calcaneal fractures.

**Methods:**

Twenty patients with Sanders type III intra-articular calcaneal fractures with a posterior subtalar articular displacement > 2 mm were included. Surgical outcomes were assessed by visual analogue scale (VAS) pain score, American Orthopaedic Foot and Ankle Society (AOFAS) hindfoot score, and calcaneal geometry, including Böhler and Gissane angles.

**Results:**

The Böhler angle, Gissane angle, and height and length of the calcaneus were increased following treatment. Based on the AOFAS score, 80% of cases had excellent or good outcomes. The mean postoperative VAS pain score was 1.6. Complications such as malunion or a screw positioning deviation occurred in 6 patients, and one patient experienced delayed wound healing. There were no wound infections.

**Conclusions:**

These results indicate that minimally invasive dual incision with mini plate internal fixation may be an effective alternative to ORIF for treating Sanders type III calcaneal fractures. Advantages include improvement of calcaneal geometry and a lower rate of wound infections.

## Background

Calcaneal fractures account for approximately 2% of all fractures, and 60 to 75% of calcaneal fractures are displaced intra-articular fractures [1]. Approximately 20% of patients with intra-articular calcaneal fractures will be unable to return to work within 1 year, which has significant social and economic consequences [2, 3]. Because intra-articular calcaneal fractures involve the subtalar joint surface, they typically cause an increase in calcaneal width and decreases in the Böhler and Gissane angles. Studies have reported that incongruence in the posterior facet of the subtalar joint and failure to restore the Böhler angle are predictors of poor outcomes after operative treatment of calcaneal fractures [4–6].

Closed reduction with percutaneous pinning is minimally invasive and is believed to result in fewer soft tissue injuries and fewer wound complications compared with open reduction [7]. Indications for closed reduction include Sanders 2C tongue-type fractures, displaced calcaneal tuberosity fractures, temporary stabilization of fractures with severe soft tissue compromise, and fractures in patients with relative contraindications to open surgery. The ideal treatment for calcaneal fractures that do not meet the criteria for closed reduction is not clear [8, 9].

Operative treatment of calcaneal fractures by open reduction and internal fixation (ORIF) has been the gold standard treatment for decades; however, ORIF is associated with complications including wound infection, wound dehiscence, flap devascularization, and injury to the sural nerve [10, 11]. The complication rate ranges from 15 to 25%, and the infection rate ranges from 0.4 to 27% [5, 12]. The management of intra-articular calcaneal fractures is further complicated by the calcaneal anatomy and local soft tissue conditions, and this is particularly true of Sanders type III fractures.

Minimally invasive internal fixation through a small tarsal sinus incision has received attention in recent years as a treatment for intra-articular calcaneal fractures [13]. While this treatment is currently accepted by many scholars as a treatment for Sanders type II intra-articular calcaneal fractures, there is disagreement as to whether the method is suitable for Sanders type III fractures [14–16]. Thus, the purpose of this study was to examine a combination of a minimally invasive dual incision with internal fixation using mini plates for the treatment of Sanders type III calcaneal fractures.

## Methods

### Patients

This study was approved by the Ethics Committee of our hospital, and all patients provided written informed consent to participate in the study and for all operative procedures performed.

Patients eligible for inclusion were those with a Sanders type III intra-articular calcaneal fracture and posterior subtalar articular displacement > 2 mm. Exclusion criteria were as follows: (1) open fractures accompanied by multiple injuries in the brain, chest, and/or abdomen; (2) computed tomography (CT) showing severe articular surface crushing that cannot be reconstructed; and (3) diabetes with blood glucose that is not well-controlled. Based on the above criteria, 20 patients received surgery and were included in the study.

### Preoperative management

All patients received CT scans of the injured foot, with a focus on the calcaneus, and also received preoperative calcaneal lateral and axial radiography. Fracture morphology and articular surface collapse were then evaluated by three-dimensional (3D) reconstruction to create an operative plan.

### Surgical procedure

In all patients, the small lateral incision was planned to begin approximately 1.0–1.5 cm below the lateral malleolus tip, with slight extension centered on the tarsus sinus. The small medial incision was planned over the posterior tuberosity three fingers breadths below the medial malleolus. After administration of anesthesia, a thigh tourniquet was placed and inflated, and the skin of surgical area was sealed with surgical film. A small medial incision was made to expose the medial wall of calcaneus and sustentaculum tali. A small lateral incision was then made through the lateral tarsal sinus to expose the lateral calcaneal wall and to distract the tendon that protects the fibula and nervi suralis. The end of calcaneofibular ligament was sharply dissected to expose the rear surface of the subtalar joint. Two 1.5-mm Kirschner wires were inserted into the talar neck and bent to retract and protect the soft tissue. At this time, the condition of the fracture and collapse of the rear surface of the subtalar joint could be directly observed. A single 4.0-mm Steinmann wire was inserted transversely into the interior side of the calcaneus through the tuberosity of the calcaneus where the bone is thick. Subsequently, another 4.0-mm Steinmann wire was inserted transversely into the interior lower part of the tibia. A bone distractor was placed at the medial side via the Steinmann wire and then gradually distracted to reset the tuberosity of the calcaneus. In the course of this process, the tuberosity shifted up, with inversion backward to the lower part.

A miniature T-shaped bone plate was placed on the medial wall of the calcaneus, and the sustentaculum tali and the calcaneus tubercle were connected using a semi-cortical technique to fix the screws. Next, the lateral part of the articular surface was carefully distracted through the lateral incision of the calcaneus. A small bone pry was used to lift the middle bone block, which was collapsed into the posterior part of the calcaneus, using the medial part of the articular surface as a reference point. Two 1.2-mm Kirschner wires were fixed to and penetrated the sustentaculum tali. Then, the 2 Kirschner wires were retraced through the medial incision until the end of Kirschner wire was level with the bone surface. After repositioning the lateral articular surface and confirming that the articular facet was flat, two 1.2-mm Kirschner wires were introduced in parallel under the extrovert articular surface.

The anterior bone mass of the calcaneus was reduced and temporarily fixed with Kirschner wires at the position of the sustentaculum tali. To restore the width of the calcaneus, sideway extrusion was performed on the lateral portion of the calcaneus. One guide wire was introduced in the calcaneal tubercle from the lateral back lower portion to the medial front upper portion until the sustentaculum tali was reached. Another guide wire was drilled through the calcaneal tubercle from the upper medial back portion to the lower lateral front portion until the forepart of the calcaneus was reached. A 7-hole 2.5-mm plastic mini locking plate was shaped, and then inserted through the small lateral incision, slide along the lower edge of the articular surface of the calcaneus, and finally placed on the lateral side of the calcaneus. Multiple screws were placed at the lower part of the posterior articular surface of the calcaneus and the anterior portion of the calcaneus, and two 5-mm fully threaded cannulated screws were inserted through the previous two guide wires. One cannulated screw was fixed from the calcaneus tubercle to the anterior part of the calcaneus, and the other one was fixed from the calcaneus tubercle to the sustentaculum tali. For patients with a tongue-type fracture, a 4.0-mm fully threaded cannulated screws were used for fixation perpendicular to the fracture line.

After confirming reduction of the fracture and the length of the internal fixtures were appropriate by fluoroscopy, the operative area was flushed with saline solution. Bone allograft (Osteolink Co., Ltd., China) was placed based on the size of bone defect after fracture restoration. The incisions were closed in layers, and one indwelling rubber drain was placed in each wound.

### Functional outcome evaluation

Postoperative patients were scheduled for re-examination at 1, 2, 3, 6, 12, 18, 24, 30, and 36 months after surgery. Healing of the incisions was observed, and any complications were addressed. The quality of fracture reduction was evaluated by radiographs and CT. The Böhler angle, Gissane angle, and the length, width, height, and articular surface steps were recorded preoperatively, postoperatively, and at the final follow-up. The American College of Foot and Ankle Surgery (AOFAS) score was used for evaluation of functional outcomes [14]. A visual analogue scale (VAS) pain score was used to evaluate all patients. Functional outcomes and complications were assessed and recorded by an independent surgeon who was unaware of the study design.

### Statistical analysis

Categorical variables were reported as number and percentage (%), and continuous variables as mean ± standard deviation. Radiographic measurements were reported as median and interquartile range (IQR). The Wilcoxon signed-rank test was used for comparisons between preoperative and postoperative variables. All statistical analyses were 2-sided, and values of *p* < 0.05 were considered statistically significant. All statistical analyses were performed using the SPSS version 22.0 software (IBM Corp, Armonk, NY).

## Results

### Patient characteristics

Twenty patients, 16 males and 4 females, with a mean age of 39.75 ± 11.59 years, received surgery and were included in the study. In 95% of the patients, the mechanism of injury was a fall from height. Seven patients had left side fractures, and 13 right side fractures. Based on Tscherne soft tissue injury score, 13 patients were closed fracture grade I, and 7 patients were closed fracture grade II. The average time between hospital admission and surgery was 6.75 ± 3.26 days. The main reasons for the extended time were soft tissue swelling, comorbidities, and in some cases patients taking anticoagulant drugs. The mean follow-up period was 41.95 ± 4.41 months (Table 1).

**Table 1:** Patient characteristics (*N* = 20)

Age, years	39.75 ± 11.59
**Sex**	
Male	16 (80.0)
Female	4 (20.0)
**Smoke**	11 (55.0)
**Mechanism of injury**	
Fall from height	19 (95.0)
Traffic accident	1 (5.0)
**Injury side**	
Left	7 (35.0)
Right	13 (65.0)
**Hospital admission to surgery time, days**	6.75 ± 3.26
**Follow-up time, months**	41.95 ± 4.41

Categorical variables were reported as number and percentage (%). Continuous variables were reported as mean ± standard deviation

### Operative outcomes

As compared to the preoperative values, the calcaneal width and articular surface decreased after surgery, whereas the Böhler angle, Gissane angle, and calcaneal height and length increased after the surgery (Table 2). The mean Böhler angle increase was 48.60°; the mean Gissane angle increase was 25.00°; the mean calcaneal height increase was 14.17 mm; the mean calcaneal length increase was 2.05 mm; the mean calcaneal width decrease was 11.11 mm; the mean articular surface decrease was 5.45 mm (all, *p* < 0.01). Importantly, there were no significant differences in the aforementioned variables between those measured postoperatively and those measured at the final follow-up (data on file).

**Table 2:** Preoperative and postoperative radiographic measurements

	Preoperative	Postoperative	Change between preoperative and postoperative measurements	*p*
**Böhler angle, °**	**−** 12.50 (21.75)	31.00 (13.25)	44.50 (22.75)	< 0.01
**Gissane angle, °**	101.50 (20.25)	123.00 (10.25)	18.50 (28.50)	< 0.01
**Height of calcaneus, mm**	32.95 (7.20)	46.25 (4.05)	12.60 (7.75)	< 0.01
**Width of calcaneus, mm**	51.90 (17.68)	41.95 (7.53)	**−** 9.30 (14.25)	< 0.01
**Length of calcaneus, mm**	79.35 (6.25)	81.50 (7.30)	1.85 (2.63)	< 0.01
**Articular surface, mm**	5.05 (2.53)	0.10 (1.13)	**−** 4.55 (2.55)	< 0.01

Data are reported as median (IQR). Preoperative and postoperative data were compared with the paired *t* test

**Fig. 1:**
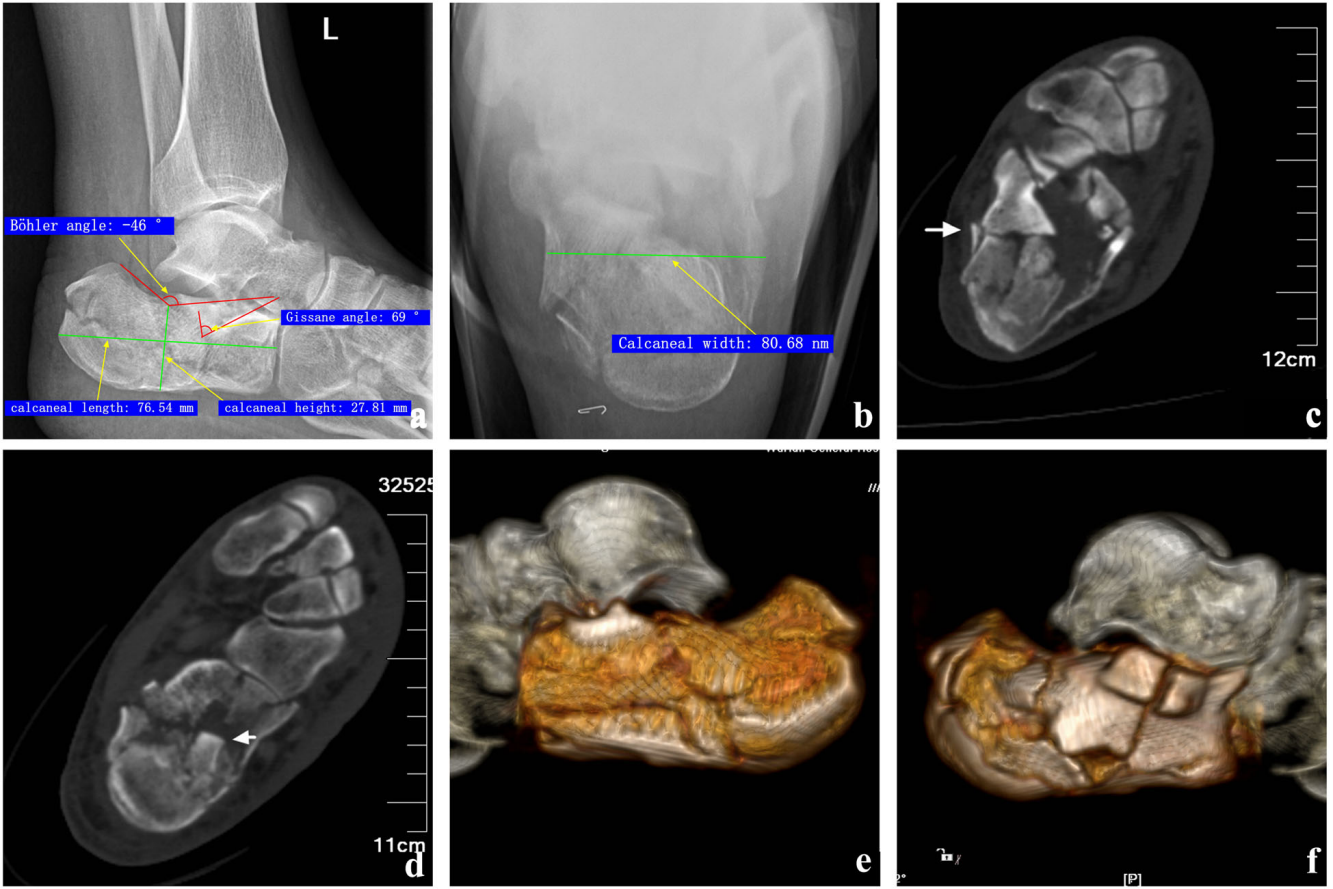
Sanders type III calcaneal fracture in a 47-year-old male. **a** Preoperative lateral axial radiograph of the calcaneus shows articular surface collapse. Böhler angle, **−** 46°; Gissane angle, 69°; calcaneal height, 27.81 mm; calcaneal length, 76.54 mm. **b** Preoperatively, the calcaneus is widened. Calcaneal width, 80.68 nm. **c**, **d** The articular surface has a comminuted fracture, and the middle mass of the bone is sunk into the calcaneus. **e**, **f** CT demonstrated calcaneal medial wall crush, and intercalary displacement of bone mass

**Fig. 2:**
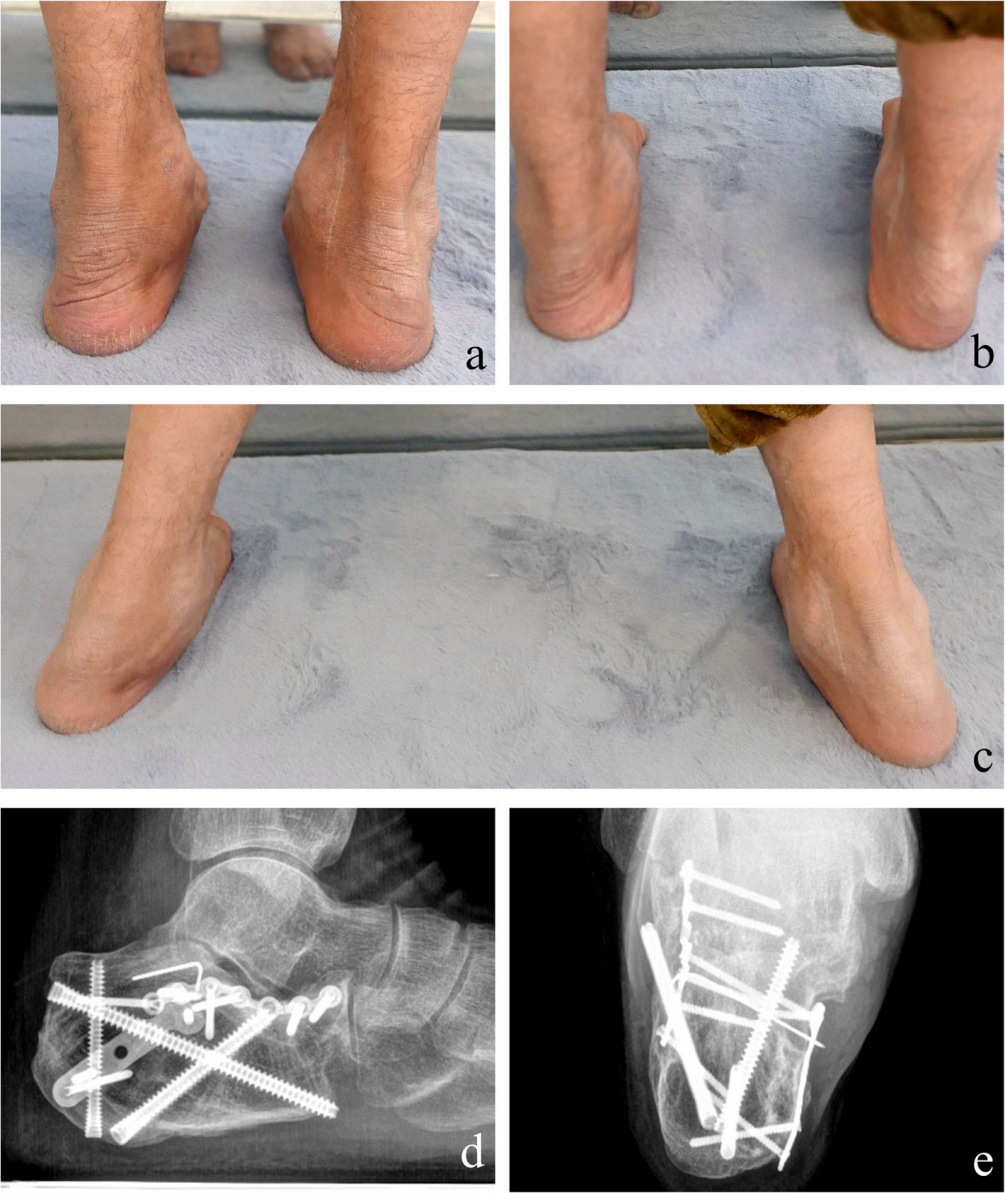
Radiographs of the patient in Fig. 1 at 40 months after surgery. **a**–**c** Postoperative view shows that the functional have returned to near-normal. **d** Calcaneal lateral axis view shows good reduction of the fractures. Bohler angle, 25°; Gissane angle, 125°; calcaneal height, 47.30 mm; calcaneal length, 77.64 mm. **e** The calcaneal width was decreased after surgery. Calcaneal width, 40.04 mm

Preoperative and 40-month postoperative radiographs, CT images, and measurement of a representative case are shown in Figs. 1 and 2. Of note, as compared to the preoperative values, the calcaneal width and articular surface decreased after surgery, whereas the Böhler angle, Gissane angle, and calcaneal height and length increased after the surgery.

### Functional outcomes

Based on AOFAS hindfoot scores, 7 patients (35%) had excellent results and 9 (45%) good results; thus, 80% of patients had good or excellent outcomes. Three (15%) patients had fair scores, and 1 (5%) patient had a poor score. The mean AOFAS score was 82.5 points (Table 3). The mean postoperative VAS pain score was 1.6 ± 1.35.

**Table 3:** Postoperative outcomes and complications

**AOFAS**	82.5 ± 11.04
Excellent (> 90)	7 (35.0)
Good (75–89)	9 (45.0)
Fair (50–74)	3 (15.0)
Poor (< 50)	1 (5.0)
**VAS pain score**	1.6 ± 1.35
**Complications**	8 (40.0)
Screw positioning deviation	6 (30.0)
Malunion	1 (5.0)
Delayed wound healing	1 (5.0)

*AOFAS* American Orthopaedic Foot and Ankle Society, *VAS* visual analogue scale. Categorical variables were reported as number and percentage (%). Continuous variables were reported as mean ± standard deviation

### Complications

There were 8 postoperative complications: 6 cases of screw positioning deviation, 1 malunion, and 1 case of delayed wound healing. There were no postoperative wound infections.

## Discussion

ORIF is the standard treatment for Sanders type II and type III fractures because it results in overall good outcomes and is cost-effective [6, 17]. However, ORIF is associated with complications such as wound infection with a reported incidence of 0.4 to 27% [5, 12], as well as wound dehiscence, flap devascularization, and injury to the sural nerve [10, 11]. This study demonstrated that Sanders type III calcaneal fractures can be successfully treated with minimally invasive dual incision and mini plate internal fixation. The method resulted in an 80% good and excellent outcome rate and a lower postoperative infection rate than ORIF, i.e., no infections in 20 patients treated.

It must be noted that the current study involved a small number of patients, and various other alternatives to ORIF have been reported with similarly good results. Several watershed studies of calcaneal fractures treated with procedures less invasive than ORIF have assessed outcomes in terms of the level of restoration achieved for aspects of the geometry of the calcaneus and the articular surface. Key geometrical parameters, including the Böhler angle, Gissane angle, and the height and length of the calcaneus are generally substantially decreased in patients with calcaneal fractures. Percutaneous techniques [7, 18, 19], in sinus tarsi versus extensile lateral approaches [14], and a minimally invasive technique using a locking plate [20] have been shown to improve or completely restore the Böhler and/or Gissane angles. Other studies have shown that certain minimally invasive techniques can improve the geometrical parameters [16, 20]. A study of Sanders type IV fractures found that the sinus tarsi approach results in improved reduction of the articular surface compared with a minimally invasive longitudinal approach [21]. Compared with the aforementioned studies, the current method resulted in greater increases in the Böhler angle, the Gissane angle, and the height and length of the calcaneus. The improvements were maintained with a mean follow-up of approximately 42 months, and good function was maintained. Thus, this technique is comparable to other alternatives to ORIF.

When comparing ORIF and minimally invasive internal fixation, studies have concluded that the treatment approach should be selected on a case-by-case basis [15]. However, the approach used in the current study represents an intermediate level of invasiveness between ORIF and minimally invasive internal fixation. Importantly, 80% of patients had good or excellent outcomes based on AOFAS hindfoot score, and there were no wound infections, which represents a major advantage of minimally invasive dual incision and mini plates internal fixation as compared with ORIF.

There are limitations of this study that should be considered. As mentioned, the number of patients was relatively small. In addition, AOFAS score is not a validated outcome measure, and VAS pain score is subjective and highly variable between cultures and societies.

## Conclusions

This study demonstrated that minimally invasive dual incision and mini plate internal fixation is an effective alternative to ORIF for Sanders type III calcaneal fractures. The method results in good functional outcomes and a lower wound infection rate than ORIF.

## Supplementary information

**Additional file 1: Figure S1.** Minimal dual-incision and mini plate internal fixation. (A, B) Incision locations are marked before surgery. (C, D) A medial small incision revealed a comminuted calcaneus inner wall, and the neurovascular tendon in the front of the incision. Both require careful protection. (E) Small lateral incision revealed a collapsed articular surface. The tendon sheath and nerves of the fibula must be protected. (F, G) General appearance of the incisions after fixation. (H, I) Intraoperative fluoroscopic images.

## Data Availability

The datasets used and/or analyzed for the current study are available from the corresponding author on reasonable request.

## Abbreviations

AOFAS: American College of Foot and Ankle Surgery
CT: Computed tomography
ORIF: Open reduction and internal fixation
