# Supplementary figures and images for: Minimally invasive dual incision with mini plate internal fixation improves outcomes over 30 months in 20 patients with Sanders type III calcaneal fractures
Source: J Orthop Surg Res. 2020 May 5;15:167. doi: 10.1186/s13018-020-01644-3 (PMC7201784; doi:10.1186/s13018-020-01644-3)

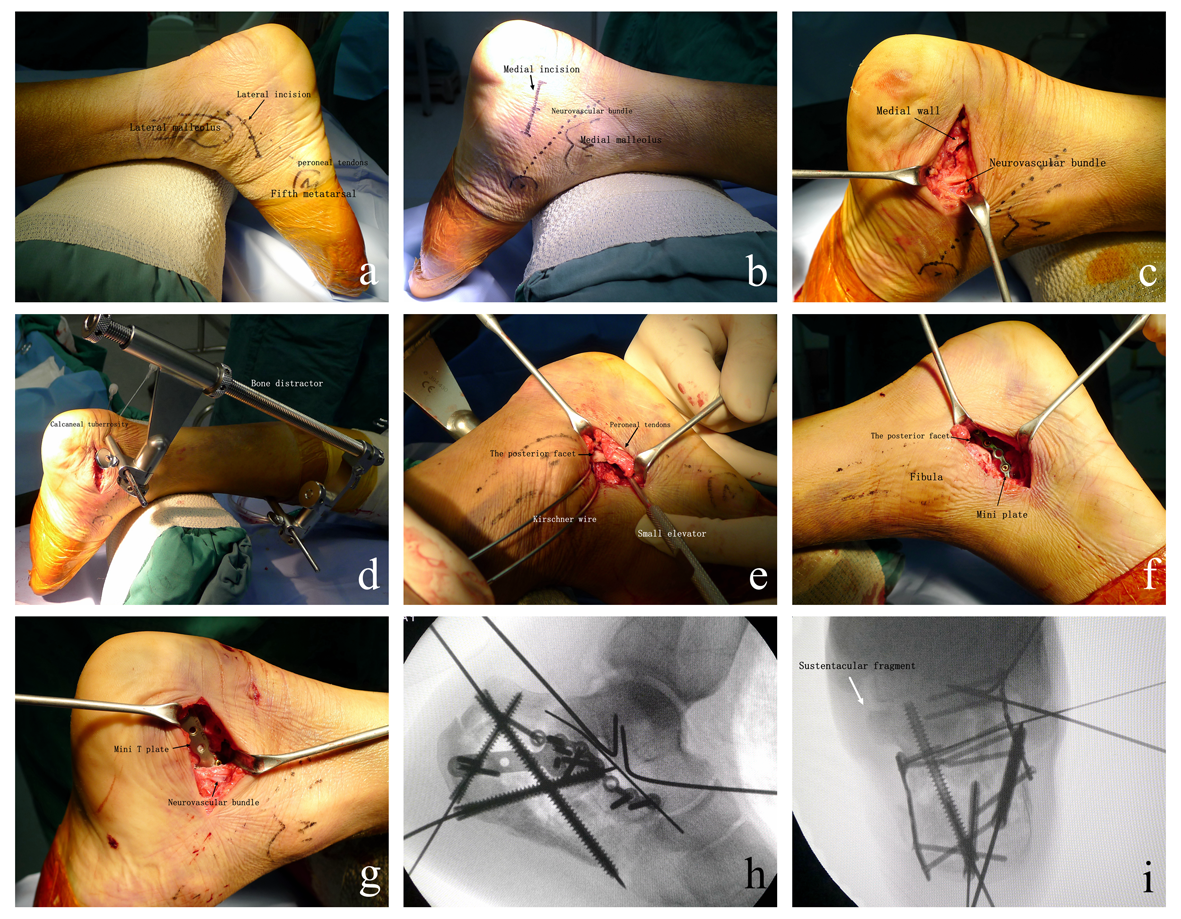

Supplement: Supplementary file 1 — Additional file 1: Figure S1. Minimal dual-incision and mini plate internal fixation. (A, B) Incision locations are marked before surgery. (C, D) A medial small incision revealed a comminuted calcaneus inner wall, and the neurovascular tendon in the front of the incision. Both require careful protection. (E) Small lateral incision revealed a collapsed articular surface. The tendon sheath and nerves of the fibula must be protected. (F, G) General appearance of the incisions after fixation. (H, I) Intraoperative fluoroscopic images. [file 13018_2020_1644_MOESM1_ESM.tif]
